# Global, regional and national disease burden of food-borne trematodiases: projections to 2030 based on the Global Burden of Disease Study 2021

**DOI:** 10.1186/s40249-024-01265-6

**Published:** 2024-12-16

**Authors:** Lu Liu, Li-Dan Lu, Guo-Jing Yang, Men-Bao Qian, Kun Yang, Feng Tan, Xiao-Nong Zhou

**Affiliations:** 1 National Key Laboratory of Intelligent Tracking and Forecasting for Infectious Diseases,National Institute of Parasitic Diseases, Chinese Center for Disease Control and Prevention (Chinese Center for Tropical Diseases Research), NHC Key Laboratory of Parasite and Vector Biology, WHO Collaborating Center for Tropical Diseases, Shanghai, People’s Republic of China; 2 Key Laboratory of National Health Commission (NHC) on Parasitic Disease Control and Prevention, Jiangsu Provincial Key Laboratory on Parasite and Vector Control Technology, Jiangsu Institute of Parasitic Diseases, Wuxi, People’s Republic of China; 3 Guizhou Provincial Center for Disease Control and Prevention, Guiyang, Guizhou People’s Republic of China; 4 School of Tropical Medicine, Hainan Medical University, Haikou, People’s Republic of China; 5 School of Global Health, Chinese Center for Tropical Diseases Research, Shanghai Jiao Tong University School of Medicine, Shanghai, People’s Republic of China; 6 Center for Global Health, School of Public Health, Nanjing Medical University, Nanjing, China; 7 Public Health Research Center, Jiangnan University, Wuxi, People’s Republic of China; 8 Chinese Center for Disease Control and Prevention, Beijing, People’s Republic of China; 9 Institute of One Health, Shanghai Jiao Tong University, Shanghai, People’s Republic of China; 10 Hainan Center for Tropical Diseases Research (Hainan Subcenter of Chinese Center for Tropical Diseases Research), Haikou, People’s Republic of China

**Keywords:** Disease burden, Food-borne trematodiases, Bayesian age-period-cohort analysis model

## Abstract

**Background:**

Food-borne trematodiases (FBTs), mainly encompassing clonorchiasis, fascioliasis, fasciolopsiasis, opisthorchiasis, and paragonimiasis, is a neglected public health problem, particularly in the WHO South-East Asia and the Western Pacific regions. This study evaluates the global, regional, and national disease burden of FBTs from 1990 to 2021 and projects trends to 2030, underscore the need for targeted prevention and control.

**Methods:**

Using the Global Burden of Disease 2021 database, the crude and the age-standardized prevalence rate (ASPR) and age-standardized prevalence disability-adjusted life years rate (ASDR) of FBTs at the global, regional and national level from 1990 to 2021 were described. The pivotal years of trend changes were identified using joinpoint regression analysis. The effects of age, period, cohort on FBTs prevalence and correlation with the sociodemographic index (SDI) was analyzed. Finally, the worldwide disability-adjusted life years (DALYs) for FBTs, projected up to 2030 using the Bayesian age-period-cohort model, were analyzed.

**Results:**

In 2021, 44,466,329 FBTs cases [95% uncertainty interval (UI): 40,017,217, 50,034,921], and 998,028 DALYs [95% UI: 569,766, 1,638,112] were estimated across 17 countries. The Western Pacific region exhibited the highest ASPR and ASDR, with the values of 1649.26 (95% UI: 1461.95, 1881.64) and 36.54 (95% UI: 19.77, 64.16), respectively. From 1990 to 2021, Lao PDR, Thailand, and the Philippines showed the most substantial declines in FBTs, while Kazakhstan had the largest average annual percentage change in DALYs (− 6.60, 95% UI: − 7.10, − 6.10). High-middle and middle SDI countries exhibited higher burden, with ASDR values of 28.03 (95% UI: 15.41, 48.73) and 16.63 (95% UI: 9.32, 27.68), respectively. The disease burden was greater among males, peaking in the 50–59 age group. The projected ASDR in 2030 is 13.10 for males and 8.40 for females.

**Conclusions:**

FBTs remain a public health threat, with the global ASDR projected to remain stable, showing only a slight decrease by 2030. Low-income countries face ambiguous mortality rates and underestimated disease burdens, highlighting the need for improved surveillance. To achieve the 2030 NTD goal, comprehensive surveillance and integrated strategies derived using a One Health approach should be prioritized to control FBTs effectively.

**Graphical Abstract:**

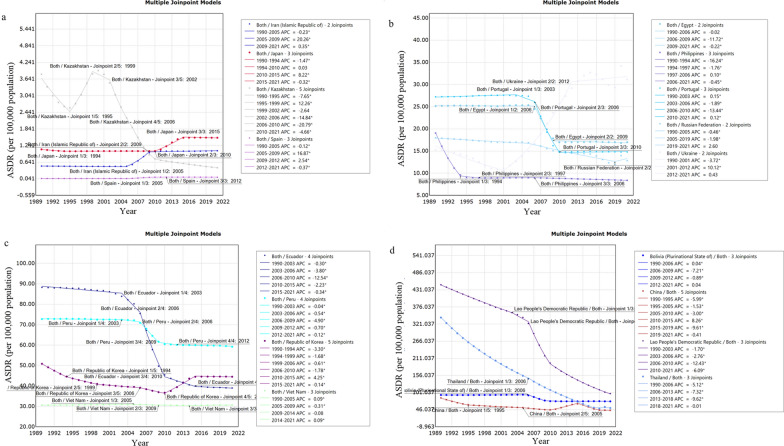

**Supplementary Information:**

The online version contains supplementary material available at 10.1186/s40249-024-01265-6.

## Background

Food-borne trematodiases (FBTs) comprise a cluster of trematode infections that represent a significant and often overlooked public health problem [1]. It was estimated that at least 99 species of foodborne trematodes can infect humans, including 15 liver flukes, 9 lung flukes, and 75 intestinal flukes in term of taxonomy [2]. FBTs have been included by the World Health Organization (WHO) in the list of 21 neglected tropical diseases (NTDs), with a focus on trematodiases infected by *Clonorchis sinensis*, *Opisthorchis felineus*, and *O. viverrini* (collectively known as small liver flukes), *Fasciola hepatica*, *F. gigantica*, and *Paragonimus* spp [3]. Human infections typically occur through ingestion of contaminated fish, crustaceans, or plants carrying the larvae of these trematodes. Infection with foodborne flukes can lead to serious pathology in the liver, biliary, lungs, and intestines system of humans, resulting in socioeconomic loss [1]. Notably, *O. viverrini* and *C. sinensis* infections have been classified as “carcinogenic to humans” (Group 1) by the International Agency for Research on Cancer (IARC) in 1994 and 2009 [4].

Since 2006, the WHO has launched an initiative to estimate the Global Burden of Disease (GBD) including FBTs using the index of disability-adjusted life years (DALYs). This initiative has addressed a significant gap in the global understanding of the burden posed by FBTs. A systematic review in 2021 found that, according to data from the WHO Global Health Observatory, 42 percent (93/224) countries reported at least one foodborne trematode infection, and 26 countries are likely co-endemic to two or more FBTs [5]. GBD 2019 estimated 3,353,7841.58 prevalent cases with FBTs and the age-standardized DALYs rate (ASDR) at 9.47 globally in 2019 [6]. Literature reports indicated that the main burden of FBTs was concentrated in WHO South-East Asia and the Western Pacific regions, particularly in the Greater Mekong region. Additionally, there were isolated areas of prevalence in other countries [5]. With the development of globalization, increased international travel, and change in culinary habits, cases of FBTs have been reported in many non-endemic countries and regions in recent years [7]. FBTs are becoming an increasingly important global health threat. The road map for NTDs 2021–2030 showed that FBTs were prevalent in 92 countries across all continents except Antarctica [3]. However, in some countries, especially in African nations, the chronic symptoms caused by FBTs were not considered a priority and were not monitored, resulting in a lack of relevant data [8]. Furthermore, the diagnosis of FBTs has low sensitivity and specificity, which means that many endemic regions may remain undiscovered, leading to a significant underestimation of the global FBTs burden. The 2021–2030 NTD road map aims to strengthen control in highly endemic areas, with a target of achieving control in 11 countries by 2030 [3].

Therefore, there is an urgent need to analyze the epidemiological characteristics of FBTs at global and national levels, develop highly sensitive and specific diagnostic techniques, and implement targeted control measures to achieve the 2030 goals. The GBD is the only global initiative that systematically and scientifically quantifies the comparative impact of health loss from all major diseases, injuries, risk factors by age, sex, and population over time[9]. This study utilizes the newly released GBD 2021 database to analyze the epidemiological characteristics of FBTs, mainly encompassing clonorchiasis, fascioliasis, fasciolopsiasis (intestinal fluke), opisthorchiasis, and paragonimiasis, at the global, regional and national levels, and project trends in disease burden up to 2030.

## Methods

### Data sources

The crude and age-standardized rates (ASRs) of prevalence, mortality, death, years of life lost (YLLs), years lived with disability (YLDs) and DALYs for 371 diseases and injuries in 204 countries and territories and 811 subnational locations from 1990 to 2021 were estimated in GBD 2021 per 100,000 population [9, 10]. The specific estimation methods for GBD 2021 study have been described in detail in previous studies [11]. In this study, “Food-borne trematodiases” was chosen for the cause, and “Prevalence”, “YLDs”, “DALYs” for the measures, “Rate (per 100,000 population)” for the metric from the database. DALYs are the combinations of YLLs and YLDs. In the database, there are no recorded death cases attributable to FBTs, thus resulting in the absence of YLLs. Consequently, the index DALY was used to represent the disease burden of FBTs, which is equivalent to the value of YLD.

The sociodemographic index (SDI) measures a country’s sociodemographic development by combining income per capita, education, and total fertility rate (TFR) [12]. The SDI ranges from 0 to 1, where 1 represents the highest socioeconomic levels. Depending on their 2021 SDI values, countries were categorized as low SDI (0–0.45), low-middle SDI (0.45–0.61), middle SDI (0.61–0.69), middle-high SDI (0.69–0.81), and high SDI (0.81–1).

### Analysis of overall temporal trends in disease burden of FBTs

The temporal trends in disease burden of FBTs on a global scale, across WHO regions, and at the national level from 1990 to 2021 were examined. The variations based on gender and specific countries were also considered. Additionally, the relationship between the DALYs of FBTs and countries with different SDI based on Pearson correlation analysis was explored.

### Joinpoint regression analysis

The pivotal years of the temporal trend in FBTs disease burden from 1990 to 2021 at the global and national level were identified using the joinpoint regression program (version 4.9.1, National Cancer Institute, USA). The analysis quantified trends using the annual percentage change (APC) and the average annual percentage change (AAPC) [13]. Calculations were based on the default options in the joinpoint program, selecting the best model with up to five joinpoint corresponding to six segments [14]. An APC or AAPC value, along with its 95% confidence interval (*CI*) greater than 0 indicate an increasing trend, whereas an APC or AAPC value with its 95% *CI* less than 0 indicates a decreasing trend.

### Age-period-cohort modelling analysis of prevalence data

An age-period-cohort model was used to assess the age, period, and cohort effects on prevalence rate of FBTs [15]. The intrinsic estimator (IE) method was used to address the dependency among age, period, and cohort by applying the principal component regression analysis, as described in previous literature [16]. The prevalence cases for FBTs and global population data were used as data inputs for the age-period-cohort model. The population aged 0–99 years was divided into twenty age groups (0–4, 5–9, …, 94–99) with a group distance of five years. Time point values (1990–1994, 1995–1999, …, 2020–024) with five-years interval were used for the period. The birth cohort was calculated as “period–age”, generating a total of twenty-six birth cohorts (1895–1899, 1900–1904, …, 2020–2024) based on seven period groups and twenty age groups (a detailed schematic is in Additional file: Table S1). Coefficients and 95% *CI* for age, period, and birth cohort were calculated using STATA 16.0 (Stata-Corp, College Station, TX), and relative risk (RR) was assessed for age, period and birth cohort-specific ratios.

### Global projection of DALYs for FBTs by Bayesian Age-Period-Cohort (BAPC) modelling up to 2030

Based on the effects of age, period and cohort on FBTs, a BAPC model was developed to project the trends of the FBTs burden up to the year 2030. The BAPC shows better coverage and precision, as demonstrated in previous studies [17, 18]. The estimated population data was obtained from the United Nations World Population Prospects 2021 Revision, categorized by year (up to 2100), age, and sex (https://poulation.un.org/wpp/Download/Standard/Population/) and the WHO World Population Data (WHO 2000–2025) standards. The calculation process utilized the “BAPC” R package and all statistical analyses were conducted using the statistical software R 4.3.1 (Lucent Technologies, Jasmine Mountain, USA). Values with *P* < 0.05 were considered statistically significant [19].

## Results

### Overall trends of diseases burden of FBTs from 1990 to 2021

In 2021, the worldwide number of FBTs cases [including clonorchiasis, fascioliasis, fasciolopsiasis (intestinal fluke), opisthorchiasis, and paragonimiasis] was 44,466,329 [95% uncertainty interval (UI): 40,017,217, 50,034,921], with an ASPR of 526.74 (95% UI: 473.70, 593.25) (Table 1). In 2021, the global DALYs for FBTs were 998,028 (95% UI: 569,766, 1,638,112), and the ASDR were 11.78 (95% UI: 6.72, 19.46), representing a reduction of 2.40 (95% UI: − 2.60, − 2.30) from 1990 to 2021. The prevalence of FBTs is higher in males than in females with 26,792,503 cases in males (95% UI: 23,935,772, 30,187,221) and 17,673,826 cases in females (95% UI: 15,992,365, 19,815,428) in 2021.

**Table 1:** Crude number and age-standardized rates (per 100,000 population) of prevalent cases and DALYs attributable to FBTs in 2021 by gender, SDI and county

	Prevalence (95% UI)		DALYs (95% UI)		AAPC in ASR (per 100,000), 1990–2021
	Counts, 2021	ASR (per 100,000), 2021	Counts, 2021	ASR (per 100,000), 2021	
Global	44,466,329 (40,017,217, 50,034,921)	526.74 (473.70, 593.25)	998,028 (569,766, 1,638,112)	11.78 (6.72, 19.46)	− 2.40 (− 2.60, − 2.30)
Males	26,792,503 (23,935,772, 30,187,221)	643.38 (574.77, 724.72)	603,639 (338,705, 1,017,290)	14.45 (8.09, 24.40)	− 2.40 (− 2.60, − 2.30)
Females	17,673,826 (15,992,365, 19,815,428)	411.46 (372.99, 460.13)	394,388 (222,202, 632,686)	9.15 (5.14, 14.77)	− 2.50 (− 2.70, − 2.30)
Low-middle SDI	1,457,798 (1,188,011, 1,766,133)	79.31 (65.26, 95.42)	30,646.04 (12,009.33, 55,897.82)	1.67 (0.67, 3.03)	− 2.10 (− 2.10, − 2.00)
Middle SDI	20,276,980 (18,284,060, 22,912,840)	749.04 (675.54, 842.62)	454,675.60 (257,265.80, 752,089.40)	16.63 (9.32, 27.68)	− 3.50 (− 3.60, − 3.40)
High-middle SDI	19,504,210 (17,339,510, 22,154,940)	1232.73 (1094.87, 1404.88)	449,489.30 (248,556.50, 757,464.30)	28.03 (15.41, 48.73)	− 1.10 (− 1.50, − 0.80)
High SDI	3,219,913 (2,896,007, 3,561,430)	227.96 (207.69, 253.15)	63,066.47 (39,296.59, 93,562.67)	4.48 (2.80, 6.73)	− 0.40 (− 0.60, − 0.10)
Bolivia (Plurinational State of)	405,366 (319,500, 502,710)	3561.36 (2825.69, 4409.68)	8316 (2127, 16,256)	72.74 (18.53, 142.69)	− 0.80 (− 0.90, − 0.70)
P.R. China	33,317,223 (29,251,039, 38,353,602)	1930.21 (1700.47, 2240.51)	768,297 (383,883, 367,826)	44.17 (22.08, 79.87)	− 1.70 (− 1.90, − 1.50)
Ecuador	272,428 (228,997, 330,395)	1517.18 (1279.45, 1835.83)	7022 (2641, 14,307)	39 (14.73, 79.48)	− 2.40 (− 2.50, − 2.30)
Egypt	783,255 (594,625, 991,988)	840.05 (659.18, 1056.57)	15,970 (4207, 32,016)	17 (4.47, 33.57)	− 1.30 (− 1.40, − 1.20)
Iran (Islamic Republic of)	46,702 (36,883, 58,508)	51.12 (40.68, 63.36)	965 (245, 1852)	1.05 (0.27, 2.03)	2.60 (2.50, 2.60)
Japan	265,865 (208,630, 331,390)	141.83 (114.15, 173.29)	3069 (1371, 5323)	1.52 (0.76, 2.54)	1.10 (1.00, 1.10)
Kazakhstan	3550 (3073, 4087)	18.83 (16.45, 21.66)	84 (42, 135)	0.44 (0.21, 0.71)	− 6.60 (− 7.10, − 6.10)
Lao People's Democratic Republic	269,178 (232,071, 308,340)	4028.84 (3494.33, 4585.05)	6360 (3498, 10,032)	97.68 (52.62, 154.47)	− 4.80 (− 4.90, − 4.70)
Peru	1,050,290 (835,766, 1,306,989)	2841.79 (2266.71, 3527.35)	21,916 (5970, 41,827)	59.20 (16.17, 113.03)	− 0.60 (− 0.70, − 0.60)
The Philippines	870,923 (675,559, 1,087,761)	809.99 (636, 1010.99)	8862 (4381, 14,831)	8.40 (4.31, 13.91)	− 2.70 (− 2.70, − 2.60)
Portugal	97,557 (73,958, 124,915)	721.43 (545.02, 922.28)	1976 (513, 3908)	14.80 (4.01, 29.77)	− 1.90 (− 2.00, − 1.90)
Republic of Korea	1,708,313 (1,504,742, 1,917,655)	2469.10 (2215.74, 2758.87)	31,373 (20,635, 45,258)	44.53 (29.12, 63)	− 0.60 (− 0.60, − 0.50)
Russian Federation	1,037,566 (904,277, 1,184,734)	576.54 (508.82, 652.78)	24,340 (12,504, 38,824)	12.85 (6.73, 20.19)	− 0.90 (− 1.30, − 0.60)
Spain	3018 (2171, 3965)	4.41 (3.22, 5.78)	66 (17, 128)	0.10 (0.03, 0.19)	2.10 (2.00, 2.20)
Thailand	1,896,042 (1,617,038, 2,222,411)	2215.61 (1911.34, 2583.69)	46,972 (22,685, 77,657)	51.44 (25.5, 81.33)	− 5.80 (− 6.10, − 5.60)
Ukraine	766,297 (651,739, 895,271)	1329.94 (1154.06, 1542.30)	18,883 (8947, 31,739)	31.15 (15.32, 49.87)	2.10 (1.70, 2.60)
Vietnam	1,665,329 (1,462,204, 1,880,106)	1569.11 (1385.34, 1766.90)	33,405 (22,107, 47,185)	30.68 (20.43, 43.28)	0

*DALYs* disability-adjusted life years, *FBTs* food-borne trematodiases, *AAPC* average annual percentage of change, *ASR* age-standardized rate

**Fig. 1:**
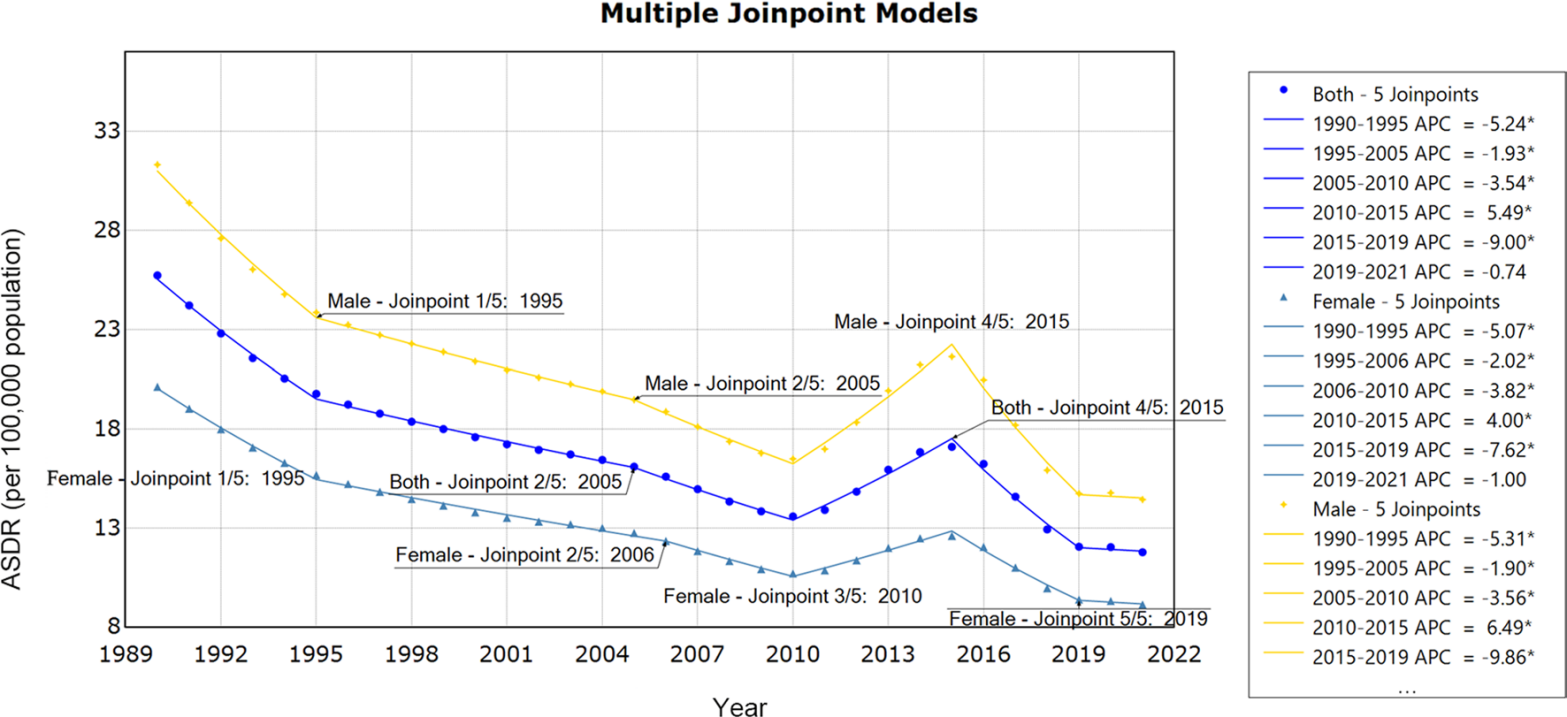
APC of ASDR for FBTs (per 100,000 population) at the global level with different gender from 1990 to 2021. Yellow line: APC of ASDR for FBTs in male; blue line: APC of ASDR for FBTs in Both gender. dark blue line: APC of ASDR for FBTs in female, * represents *P* < 0.05, *APC* annual percentage change, *ASDR* age-standardized DALY rate, *FBTs* food-borne trematodiases

Joinpoint regression analysis revealed substantial changes in the DALYs trends of worldwide FBTs in 1995, 2005, 2010, 2015, 2019. Notably, a significant decline was observed during four periods: from 1990 to 1995 (AAPC = − 5.24, 95% *CI*: − 5.60, − 4.90, *P* < 0.05), from 1995 to 2005 (AAPC = − 1.93, 95% *CI*: − 2.10, − 1.80, *P* < 0.05), from 2005 to 2010 (AAPC = − 3.54, 95% *CI*: − 3.90, − 3.10, *P* < 0.05), from 2015 to 2019 (AAPC = − 9, 95% *CI*: − 9.60, − 8.40, *P* < 0.05). However, a sudden increase in the ASDR of FBTs was observed from 2010 to 2015 (AAPC = 5.94, 95% *CI*: 5.00, 5.90, *P* < 0.05). The increase in ASDR for males (AAPC = 6.49, 95% *CI*: 6.10, 6.90, *P* < 0.05) was faster than that for females (AAPC = 4.00, 95% *CI*: 3.40, 4.60, *P* < 0.05) from 2010 to 2015 (Table 1, Fig. 1).

### Geographical variation and temporal trends in the diseases burden of FBTs at regional and national levels

**Fig. 2:**
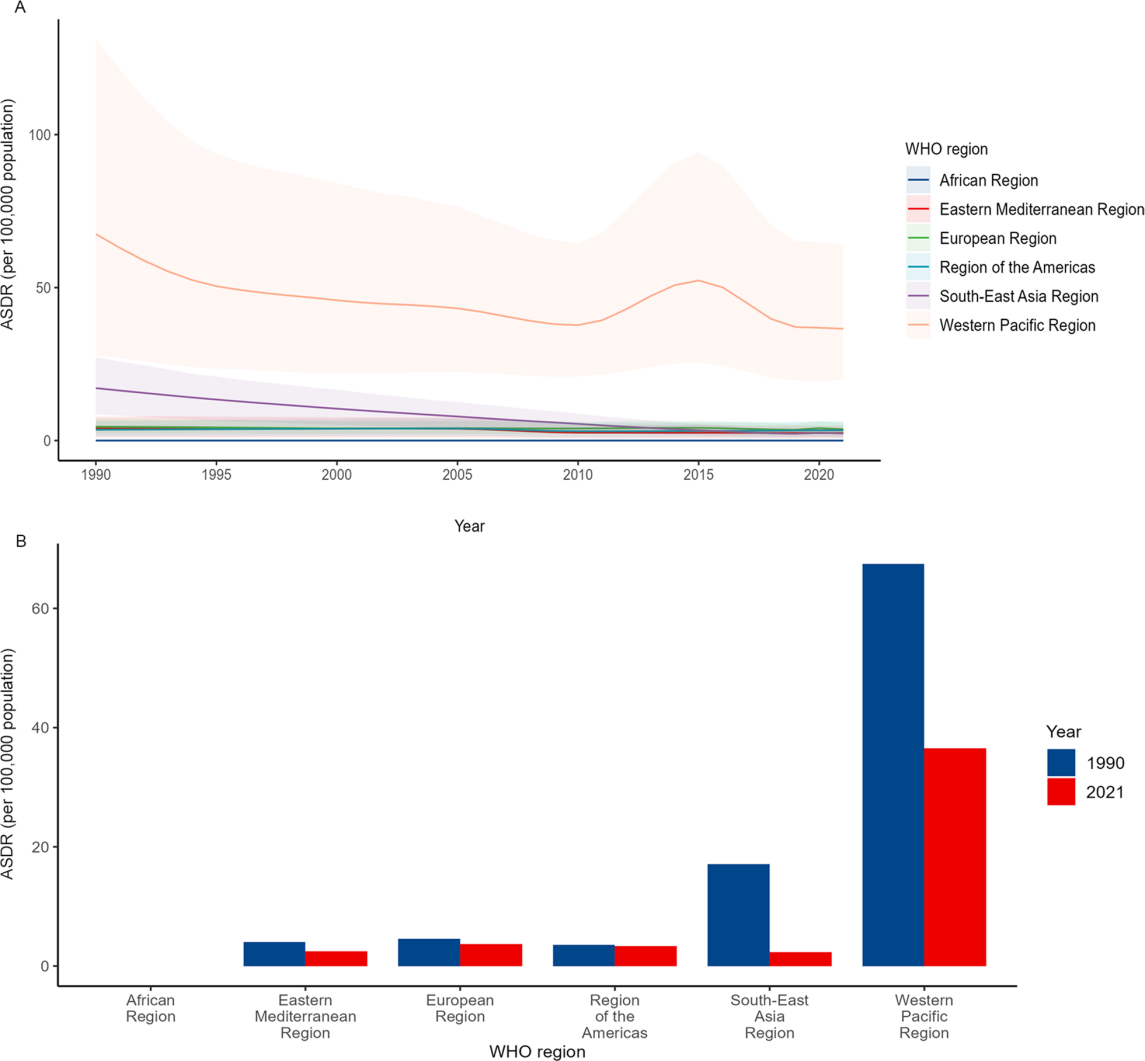
Temporal variation in ASDR of FBTs (per 100,000 population) at the WHO regional level. **A** ASDR of FBTs at WHO regional levels from 1990 to 2021, **B** ASDR of FBTs at the WHO regional level in 1990 and 2021. *ASDR* age-standardized DALYs rate, *FBTs* food-borne trematodiases

The geographical distribution of AAPC for DALYs from 1990 to 2021 is shown in Table 1, Fig. 2 and Additional file: Table S1. The Western Pacific Region consistently has the highest number of cases from 1990 (40,392,544 cases) to 2021 (38,096,830 cases), showing fluctuations, particularly a significant dip around 2005, followed by an increase from 2010 to 2015, and a decline afterward. (Fig. 2A, and Table 1). Compared to 1990, the age-standardized rates of prevalence and DALYs have decreased in all regions. Among the six regions, South-East Asia region experienced the greatest decrease, dropping from 7,603,462 cases in 1990 to 1,896,042 cases in 2021, with a decrease rate of 75.00% (Fig. 2B). However, compared to 1990, the crude cases of prevalence of FBTs in 2021 increased by 58.05% in the Region of the Americas, 49.51% in the Eastern Mediterranean Region, and 0.85% in the European Region, respectively.

As is shown in Fig. 2B, the region with the second highest ASDR ranking changed from the South-East Asia region in 1990 to the European region in 2021.

**Fig. 3:**
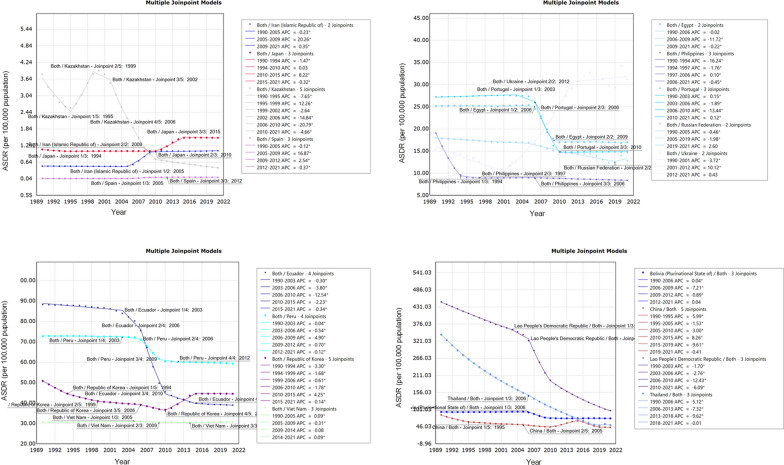
APC of ASDR for FBTs (per 100,000 population) at the country level from 1990 to 2021. *APC* annual percentage change; * represents *P* < 0.05, *ASDR* age-standardized DALYs rate, *FBTs* food-borne trematodiases

When stratified by individual nations, it was found that 17 countries have FBTs cases world widely. In 2021, the four countries with the highest number of cases are P.R. China, Thailand, Republic of Korea and Vietnam, which together account for 84.74% of the global FBT cases. Meanwhile, six countries had DALYs greater than 40, listed from highest to lowest: Lao People’s Democratic Republic (Lao PDR), Bolivia (Plurinational State of), Peru, Thailand, Republic of Korea, and P.R. China. As is shown in Table 1, from the overall trend between 1990 and 2021, 13 countries showed a decrease in the AAPC of DALYs of FBTs from 1990 to 2021, with the largest decrease in Kazakhstan (AAPC = − 6.60, 95% *CI*: − 7.10, − 6.10, *P* < 0.05). As shown in Fig. 3, between 2006 and 2010, the significant phase of decline (*P* < 0.05) in ASDR for FBTs occurred in Kazakhstan (APC = − 20.80, 95% *CI*: − 22.40, − 19.20, *P* < 0.05), Ecuador (APC = − 12.50, 95% *CI*: − 13.00, − 12.00, *P* < 0.05), Portugal (APC = − 13.40, 95% *CI*: − 13.60,− 13.20, *P* < 0.05), Lao PDR (APC = − 12.40, 95% *CI*: − 12.80, − 12.10, *P* < 0.05). The largest increase in the ASDR for FBTs from 1990 to 2021 was in Iran (Islamic Republic of) (AAPC = 2.60, 95% *CI*: 2.50, 2.60, *P* < 0.05), followed by Ukraine (AAPC = 2.10, 95% *CI*: 1.70, 2.60), Spain (AAPC = 2.10, 95% *CI*: 2.00, 2.20, *P* < 0.05) and Japan (AAPC = 1.10, 95% *CI:* 1.00, 1.10, *P* < 0.05). Notably, from 2001 to 2012, a rapid and sustained increased in the ASDR was observed in Ukraine (APC = 10.12, 95% *CI*: 9.20, 11.00,* P* < 0.05), continuing an upward trend after 2012. An increase occurred in the ASDR of FBTs in Republic of Korea from 2010 to 2015 (APC = 4.20, 95% *CI*: 4.10, 4.40,* P* < 0.05).

### National burden trends for FBTs ASDR among SDI quintiles

**Fig. 4:**
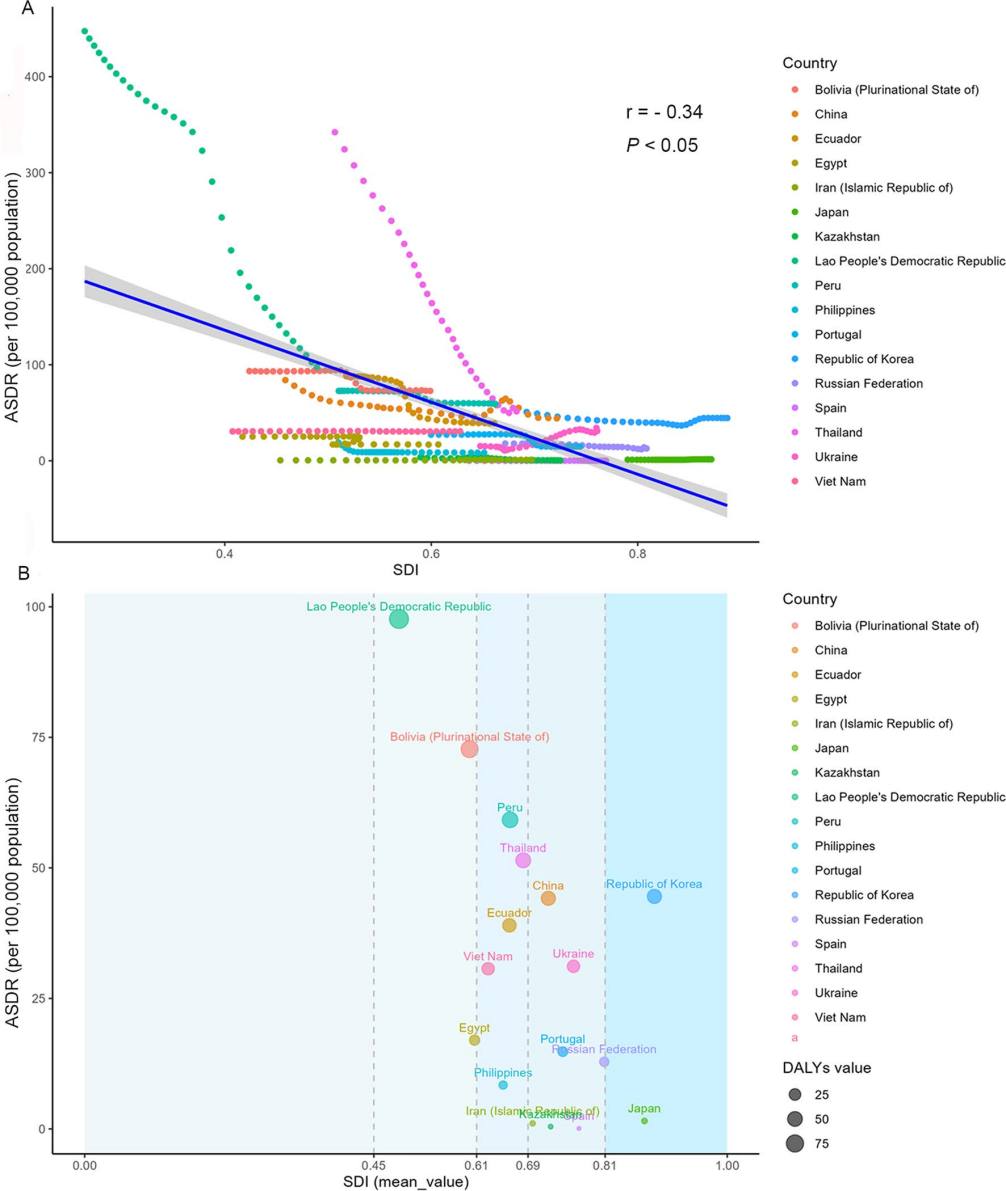
ASDR of FBTs and SDI by country from 1990 to 2021. ASDR of FBTs per 100,000 population (**A**) and SDI for 17 countries from 1990 to 2021 and (**B**) in 2021 is shown. Points represent 1 year increment from 1990 to 2021. The blue line represents the correlation between ASDR and SDI, with a correlation coefficient of − 0.34 (*P* < 0.05). *ASDR* age-standardized DALY rate, *FBTs* food-borne trematodiases, *SDI* sociodemographic index

Among the 17 countries with ASDR association from 1990 to 2021, a negative association was found among ASDR and SDI (r = − 0.34, *P* < 0.05) (Fig. 4A). DALYs declined in many countries as SDI increased, except in certain countries like Ukraine and Republic of Korean, where the ASDR increased with higher SDI. The top two countries with the highest DALYs, Lao PDR and Bolivia (Plurinational State of), have low-middle SDI. Regions with middle SDI (28.03; 95% UI: 15.42, 48.73) and high-middle SDI (16.64; 95% UI: 9.32, 27.68) had higher ASDR than other regions (Fig. 4B, Table 1).

### Age-period-cohort effects on prevalence rate of FBTs

**Fig. 5:**
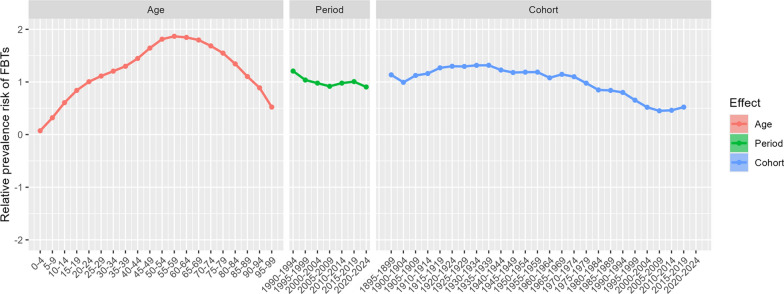
Effects of age, period and cohort on the prevalence of FBTs globally

The effects of age, period and cohort on the prevalence rate of FBTs were explored based on age-period cohort analysis (Fig. 5). The prevalence of FBTs increased with age, reaching a peak in the 55–59 age group, then decreased. The period effect curve shows a slight downward trend. The cohort effect reveals that cohorts born before 1975–1979 have a higher risk of disease, which then declines, but the risk starts to increase again for cohorts born after 1995–1999 (Additional file: Table S2).

### Projections of FBTs burden from 2022 to 2030

**Fig. 6:**
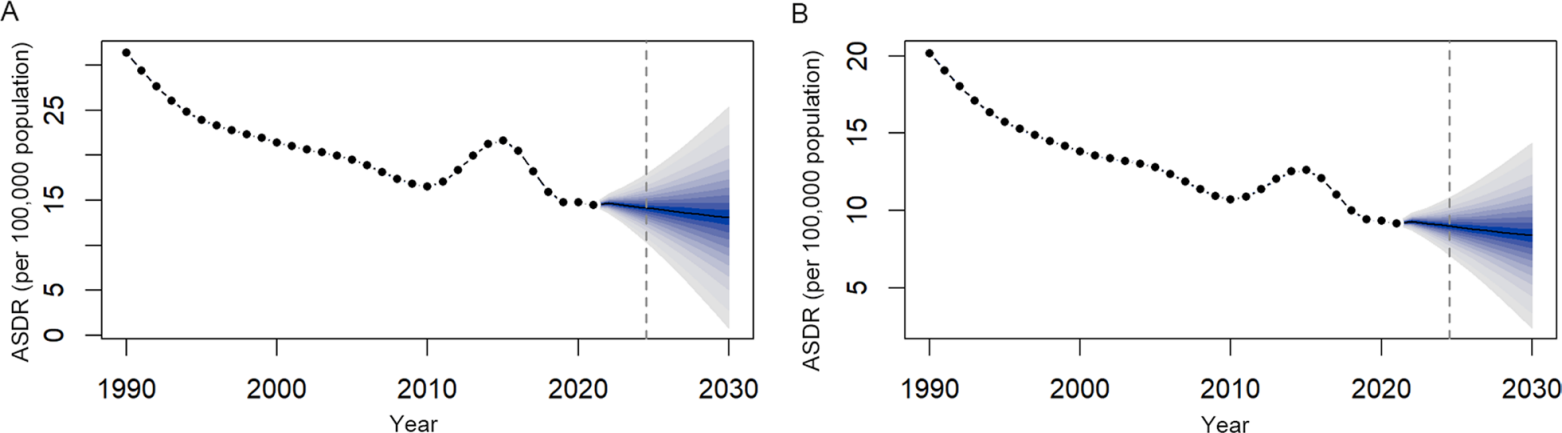
Prediction of ASDR for FBTs worldwide from 2022 to 2030 in males (**A**) and females (**B**) based on BAPC models. **A** ASDR for FBTs worldwide in males, **B** ASDR for FBTs worldwide in females. *ASDR* age-standardized DALY rate, *FBTs* food-borne trematodiases, *BAPC* Bayesian age-period-cohort

Generally, the ASDR is projected to remain stable with a slight decline in the coming years based on the BAPC model (see Fig. 6). The ASDR in males is predicted to decrease to approximately 13.10 in 2030 (Fig. 6A). Similarly, the ASDR in females is predicted to decrease slightly after 2021, reaching approximately 8.40 in 2030 (Additional file: Table S3).

## Discussion

FBTs are zoonotic diseases intricately linked to food safety[19]. The transmission of FBTs is significantly influenced by human behaviors in endemic regions, particularly in food production, preparation, and consumption [2]. The prevalence of FBTs is also influenced by ecological and environmental factors, including poverty, pollution, and population dynamics [20]. This study, covering 32 years, estimates the global disease burden and highlights variations across populations over time. It also identifies key years of significant changes in disease trends.

### The disease burden of FBTs remains significant and neglected

In 2021, the global ASPR of FBTs was 526.74, with a wide uncertainty interval of 473.70 to 593.25, requiring careful interpretation due to estimation uncertainties. Compared to 1990, overall prevalence and DALYs have decreased, indicating effective disease control efforts, primarily through chemotherapy [3, 21]. Education programs for schoolchildren, and mass drug administration (MDA) for clonorchiasis in P.R. China have reduced infection risk [21]. However, some countries show increasing prevalence trends. For instance, Ukraine, Vietnam, the Republic of Korea, Iran, the Russian Federation, and Portugal have seen an upward trend in disease burden from 2012 to 2021, though the APC was not statistically significant. Joinpoint regression analysis indicates pivotal years of trend changes in FBT burden in 1995, 2005, 2010, and 2019, likely linked to enhanced WHO initiatives and national surveillance of FBTs. The WHO has estimated DALYs for 14 groups of NTDs since 2005 [3]. Interestingly, the ASPR and ASDR of FBTs in Peru, Ecuador, Portugal, Russia, Lao PDR, and Bolivia have decreased around 2005. The increasing trend from 2010 is related to improvements in diagnostic technology and increased examination frequency [22].

### Heterogeneity in geographic distribution of FBTs

Geographically, FBTs are nearly universally distributed, with primary concentrations in the WHO Western Pacific and South-East Asia regions, where dietary habits conducive to FBTs are deeply entrenched and resistant to rapid change, consistent with previous literature [22, 23]. It was suggested that there is a need to strengthen the focus and intensity of FBTs monitoring in the Western Pacific region in the future. In this study, among the six countries with DALYs greater than 40, four were in Asian and two in South America. Previous research indicates that clonorchiasis and opisthorchiasis are primarily found in Asia, whereas paragonimiasis occurs in Africa, Asia, and Latin America[24, 25]. In the Greater Mekong Subregion, the highest pooled prevalence of opisthorchiasis was observed in Lao PDR and Thailand [18]. For clonorchiasis, the prevalence was 25.33% (95% *CI*: 18.32%, 32.34%), with Guangxi, P.R. China, exhibiting the highest rate at 26.89% (95% *CI*: 18.34%, 35.43%) [24, 25]. Previous studies showed that *Fasciola hepatica* is widespread, impacting numerous countries globally, with *F. gigantica* distribution restricted to Africa and Asia [5, 7]. In this study, no FBTs cases were reported in the African region, but previous studies reported that human paragonimiasis is present in Western Africa with a prevalence ranging from 2.00 to 31.00% [26]. The highest study prevalence of paragonimiasis from a scoping review was reported in Cameroon at 14.90% [5]. Other endemic countries with local FBTs cases, such as Cambodia with *O. viverrini* infection and fascioliasis, were not incorporated into the GBD 2021 database [27]. Several species of lung flukes in the genus *Paragonimus* are local FBTs in the USA, where an endemic cycle with various local snails and crustaceans serving as intermediate hosts exists [28]. In conclusion, the distribution range of FBTs described in the GBD 2021 has been underestimated.

### Socio-demographic factors attributed to FBTs should be further explored

The study underscores significant disparities among ASDR and SDI. In regions with middle SDI, poor healthcare access, limited access to safe water, and poor personal and environmental sanitation increase the likelihood of FBT transmission [29]. However, high SDI index countries are not free from FBTs risks, for example, Europe reports more imported fascioliasis, opisthorchiasis cases due to international travel [30]. The SDI in Vietnam increased from 0.41 in 1990 to 0.63 in 2021, but the ASDR of FBTs in Vietnam has not shown significant changes over time. This indicates that economic development may not have significantly impacted local dietary habits and disease prevention measures. Consequently, health policymakers should prioritize socioeconomic factors and tailor interventions accordingly across different SDI regions. Gender analysis reveals higher prevalence rates among males, attributed to dietary habits exposing them more to trematode-contaminated foods and cultural dietary practices [31, 32]. The higher prevalence of opisthorchiasis in males is related to the significantly higher consumption of high-risk foods like *koi pla* and *pla som* among males [32]. Adults aged 55–59 years are the most affected age group, which is not entirely consistent with previous studies. Field surveys showed that adults aged 30–54 years in P.R. China [33, 34] or 41–60 years in Lao PDR [35] were the most affected age groups for clonorchiasis or opisthorchiasis, but young children were the most affected age group for paragonimiasis [36].

### Limitations of the study

The study has several limitations, including reliance on the GBD database, which aggregates national and regional data. This aggregation may affect data integrity, timeliness, and quality, particularly in low-income settings. Additionally, GBD 2021 database only covers four types of FBTs including clonorchiasis, fascioliasis, fasciolopsiasis (intestinal fluke), opisthorchiasis, and paragonimiasis, which limits its overall scope. While major FBTs are considered in the GBD, intestinal FBTs such as echinostomiasis and gastrodiscoidiasis are often neglected among NTDs and are mainly confined to certain regions of Asia [37]. Moreover, the study does not differentiate the burden by specific trematodiases types, potentially obscuring epidemiological nuances. The surveillance and collection of epidemiological data on FBTs may not be timely enough, affecting the comprehensive understanding of disease incidence. The true prevalence of these infections remains unclear, necessitating further systematic helminthological investigations to ascertain their true impact on human health.

### Prevention and control strategies derived from the One Health approach should be addressed to combat FBTs

The magnitude of the public health problem posed by these infections remains largely unknown due to insufficient information on their geographical distribution and the populations affected or at risk. From a control perspective, this issue is inadequately addressed or entirely unaddressed in most countries. Reinfection was common due to the difficulty in changing deeply rooted cultural food habits. Beyond human dietary habits, the complex life cycle of foodborne trematodes, involving snails, fish, plants, and animals makes interrupting the transmission process highly challenging [38]. Health education programs that targeted primary students were possibly more effective at promoting and maintaining changed behavior in the future communities [21]. Access to sanitation was also reported to be protective, as this reduced environmental contamination and infection intensity of FBTs [5]. The success of the Lawa Model in combating opisthorchiasis and fascioliasis endemic areas of Northern Bolivian Altiplano demonstrated the comprehensive effectiveness of the One Health approach [39]. The One Health paradigm, which includes integrated programs encompassing mass drug administration, improved water, sanitation, hygiene (WASH), community health education, surveillance, and veterinary public health interventions, is necessary to successfully combat FBTs among the poor [40]. These approaches should also be incorporated into Universal Health Care programs to guarantee fair implementation of the NTD road map by 2030 [41].

## Conclusions

This study indicated that FBTs continue to pose a threat to public health due to the global ASDR of FBTs is projected to remain stable with a slight decrease up to 2030.  The ambiguous mortality rates and prevalence suggest a significantly underestimated disease burden globally, especially in middle and high-middle SDI regions. The infection rate and intensity of specific member of FBTs need to be more clearly defined. To further mitigate the disease burden of FBTs and achieve the NTD control goal by 2030, surveillance and control programs for FBTs at global, regional and national levels should be continuously implemented through a One Health approach.

## Supplementary Information

Supplementary Material 1. Table S1. Crude number and age-standardized rate of prevalence and DALYs for FBTs in 1990 and 2021 at the WHO regional level. *DALYs* disability-adjusted life years, *FBTs* food-borne trematodiases. Table S2. The age, period, and cohort effect on the global prevalence rate of FBTs. *FBTs* food-borne trematodiases. Table S3. Projection of DALYs for FBTs in males and females up to 2030. *DALYs* disability-adjusted life years, *FBTs* food-borne trematodiases.

## Data Availability

All data are open-access and can be available at Global Health Data Exchange query tool (http://ghdx.healthdata.org/gbd-results-tool).

## Abbreviations

ASR: Age-standardized rate
ASPR: Age-standardized prevalence rate
ASDR: Age-standardized DALYs rate
APC: Annual percentage change
AAPC: Average annual percentage change
BAPC: Bayesian age-period-cohort analysis
*CI*: Confidence intervals
DALYs: Disability-adjusted life years
FBTs: Foodborne trematodiases
GBD: Global burden of disease
IARC: International Agency for Research on Cancer
IE: Intrinsic estimator
MDA: Mass drug administration
NTDs: Neglected tropical diseases
RR: Relative risk
SDI: Sociodemographic index
TFR: Total fertility rate
UI: Uncertainty interval
WHO: World Health Organization
WASH: Water, sanitation, hygiene
YLLs: Years of life lost
YLDs: Years lived with disability
